# Modeling inner hair cell ribbon synapses: response heterogeneity and efficiency of sound encoding in an idealized biophysical model

**DOI:** 10.1186/1471-2202-14-S1-P420

**Published:** 2013-07-08

**Authors:** Mantas Gabrielaitis, Nikolai M Chapochnikov, Tobias Moser, Fred Wolf

**Affiliations:** 1 Department of Nonlinear Dynamics, Max Planck Institute for Dynamics and Self-Organization, Goettingen 37077, Germany; 2 InnerEarLab, Department of Otolaryngology, University of Goettingen, Medical School, Goettingen 37075, Germany; 3 Bernstein Center for Computational Neuroscience, Max Planck Institute for Dynamics and Self-Organization, Goettingen 37077, Germany

## 

Heterogeneity of neuronal responses is abundant in sensory systems. For example, spiral ganglion neurons (SGN), which are postsynaptic to mechanosensory inner hair cells (IHCs), show high variability in spontaneous and sound-driven discharge [1]. Recent experimental evidence implicates heterogeneity of the molecular organization of the IHC presynaptic active zone as a possible mechanism of the apparent SGN response heterogeneity to auditory stimuli [2,3]. It is well known that response heterogeneity in sensory systems results in a higher capacity to cover a broad range of stimulus levels and temporal features [4,5]. However, the molecular organization principles leading to heterogeneous presynaptic responses that are shaped for optimal stimulus encoding are not well understood.

In this work, we approached this general question by studying IHC synapses in the framework of an analytically tractable biophysical model of a presynaptic active zone. This model includes vesicle docking sites each modeled as a two-state Markov chain, one state being empty and the other ready to release a docked vesicle. Vesicle release is driven by a population of presynaptic two-state Ca^2+ ^channels with voltage-dependent gating rates. The vesicle fusion rate is proportional to the calcium concentration [Ca^2+^] at the release site. We approximated [Ca^2+^] changes as instantaneous upon the Ca^2+ ^channel opening and closing, and set the spatial profile as a linear approximation of buffered diffusion. The recovery rate of empty docking sites was assumed to be constant. As a quantitative response characteristic of the synapse we considered adapted rate-level (RL) functions, one of the experimentally most studied response features, of SGN. We analyzed the temporal pattern of release events at the synapse as a proxy of the SGN spike pattern, which is justified by experimental evidence [3]. To assess the stimulus encoding quality, mutual information based on either stationary inter-release-interval or release event count was estimated.

We found that the model is capable of reproducing the SGN RL function heterogeneity semi-quantitatively including the experimentally known correlations between the activation thresholds, spontaneous and maximum rates, and dynamic ranges. These experimental requirements partially constrain the active zone organizations. Nevertheless, various parameter sets can result in similar RL functions. For example, the shift of the stimulus dependence of RL functions was achieved either by shifting the voltage dependence of Ca^2+ ^channel activation curves, changing release site recovery rates, or increasing Ca^2+ ^binding rates or [Ca^2+^] levels at the release sites. Changes in RL function slopes resulted either directly from changes in slopes of Ca^2+ ^channel voltage activation curves or varying levels of heterogeneity among different release sites of the same active zone. In spite of the relative freedom of the parameter choice in reproducing the main effects, some of the choices resulted in more efficient stimulus encoding, than the others. We discuss information-transfer-optimal organizations with particular emphasis on the biologically most plausible active zones that consist of single channel driven release sites.
